# Desoxyrhapontigenin attenuates neuronal apoptosis in an isoflurane-induced neuronal injury model by modulating the TLR-4/cyclin B1/Sirt-1 pathway

**DOI:** 10.1186/s13568-020-01105-4

**Published:** 2020-09-30

**Authors:** Feng Liang, Xin Fu, Yunpengfei Li, Fanglei Han

**Affiliations:** Department of anesthesiology, China-Japan Union Hospital of Jilin University, Changchun, 130021 Jilin China

**Keywords:** Desoxyrhapontigenin, Neuronal injury, Isoflurane, Apoptosis, TLR-4, Sirtuin

## Abstract

This study investigated the protective effect of desoxyrhapontigenin (DOP) against isoflurane (ISF)-induced neuronal injury in rats. Neuronal injury was induced in pups by exposing them to 0.75% ISF on postnatal day 7 with 30% oxygen for 6 h. The pups were treated with DOP 10 mg/kg, i.p., for 21 days after ISF exposure. The protective effect of DOP was estimated by assessing cognitive function using the neurological score and the Morris water maze. Neuronal apoptosis was assessed in the hippocampus using the TUNEL assay, and protein expression of caspase-3, Bax, and Bcl-2 was measured by Western blotting. The levels of cytokines and oxidative stress parameters were assessed by ELISA. Western blotting and RT-PCR were performed to measure the expression of NF-kB, TLR-4, Sirt-1, and cyclin B1 protein in the brain. The cognitive function and neurological function scores were improved in the DOP group compared with the ISF group. Moreover, DOP treatment reduced the number of TUNEL-positive cells and the expression of caspase-3, Bax, and Bcl-2 protein in the brains of rats with neuronal injury. The levels of mediators of inflammation and oxidative stress were reduced in the brain tissue of the DOP group. Treatment with DOP attenuated the protein expression of TLR-4, NF-kB, cyclin B1, and Sirt-1 in the brain tissue of rats with neuronal injury. In conclusion, DOP ameliorates neuronal apoptosis and improves cognitive function in rats with ISF-induced neuronal injury. Moreover, DOP treatment can prevent neuronal injury by regulating the TLR-4/cyclin B1/Sirt-1 pathway.

## Introduction

Anaesthetics are used to relieve pain to enable surgery and medical procedures (Lee 2017). Anaesthetics affect learning and memory in the developing brain by inducing neuronal apoptosis (Wu et al. 2019). At equal doses, isoflurane (ISF) damaged the brain cells of neonatal rats more than did sevoflurane (Liang et al. 2010). ISF enhances neuronal apoptosis by altering the mitochondrial membrane (Zhang et al. 2010). The Bcl-2/Bax ratio plays an important role in maintaining the integrity of the mitochondrial membrane (Wang et al. 2016). ISF has also reported to enhances apoptosis and alters the function of glial cells such as astrocytes, microglia and oligodendrocytes which contributes in the neuronal injury and altered cognitive functions (Bordone et al. 2019). Literature also reveals that ISF administration induced neuronal apoptosis by activation the microglias such as astrocytes and oligodendrocytes by altering the gene expression of CD86 in the developing brain, which further leads to induction of brain injury and reduction in the cognitive functions (Broad et al. 2016). Moreover, ISF reduces neuronal growth because it interferes with astrocyte function by reducing the levels of brain-derived neurotrophic factor (Ryu et al. 2014), which enhances neuronal growth and maturation by enhancing the expression of neuronal glucose transporter GLUT3, thereby increasing glucose utilization (Fuente-Martín et al. 2012). In a murine model, ISF exposure enhanced the expression of caspase-3 and deposition of β-amyloid peptide (Aβ), which contribute to the apoptotic pathway (Jiang and Jiang 2015). Anaesthetics enhance neurodegeneration by increasing proinflammatory cytokine levels (Tang et al. 2011). Pro-inflammatory cytokines such as interleukins (ILs) and tumor necrosis factor (TNF)-α contribute to the nuclear factor-κB (NF-κB) dependent signalling pathway, which causes cognitive impairment (Liu et al. 2017). Cerebral ischemia limits the oxygen supply to brain cells (Broad et al. 2016). Glial cells play a central role in this response, which is mediated by the TLR-4 pathway and apoptosis-associated factors, such as p53 and NF-kB (Lei et al. 2019). Moreover, in cerebral ischemia, the TLR-4 pathway is regulated by NF-kB, and a reduction in TLR-4 expression decreases inflammation in ischemic stroke (Ou et al. 2014). Thus, ischemia might be managed by targeting the TLR-4 pathway. Sirtuin (Sirt)-1 regulates cellular function and autophagy (Li et al. 2017). Activation of TLR-4 downregulates Sirt-1 expression in neuronal injury (Pucci et al. 2000). Cyclin B1 is also downregulated in inflamed tissues, which further contributes to the activation of apoptosis (Venkatesan et al. 2016).

Secondary metabolites isolated from plants have potential for treating chronic disorders, including neurodegenerative disorders. The stilbene desoxyrhapontigenin (DOP) has been isolated from *Rheum undulatum* (Choi et al. 2014a) and found to have potent antioxidant and anti-inflammatory properties (Choi et al. 2014b). DOP prevents lung injury by regulating Nrf2 and ameliorates inflammation via MAPK and NF-kB signalling (Gomes et al. 2018). Other properties of stilbenes include prevention of mitochondrial damage by modulating the expression of Sirt-1 (Qin et al. 2017). Therefore, this study examined the preventive effects of DOP on neuronal apoptosis in ISF-induced neuronal damage.

## Materials and methods

### Animals

Sprague-Dawley rat pups on postnatal day 1 were housed under a 12 h light/dark cycle at 25 ± 3 °C and 60 ± 5% humidity. The animal experiments were approved by the institutional animal ethics committee of China–Japan Union Hospital, Jilin University, China (IAEC/CJUH-JU/2018/09).

### Chemicals

DOP was purchased from Sigma Aldrich (St. Louis, MO, USA). Enzyme-linked immunosorbent assay (ELISA) kits were purchased from R&D Systems (Minneapolis, MN, USA). Antibodies used for Western blotting were obtained from Thermo Fisher Scientific (Wilmington, DE, USA).

### Experimental protocol

The pups were separated into three groups of 15 each and subjected to one of three treatments. Normal controls were allowed to grow normally. The ISF group was exposed to 0.75% ISF on postnatal day 7 with 30% oxygen for 6 h. The DOP group was also exposed to the same ISF treatment and then given DOP (10 mg/kg, i.p.) for 21 days. After 21 days, behavioural testing was performed. Then, the animals were sacrificed, and the hippocampus was removed and stored at − 80 °C until further use.

### Assessment of neurological function

Animals were assigned a neurological score, the highest score being 20 points, based on motor and behavioural findings, including level of consciousness, climbing ability, extremity tonus, walking, posture, and response to nociceptive stimuli.

### Morris water maze test

Cognitive function was evaluated at the end of the treatment protocol using the Morris water maze test (Kim and Lee 2014). Spatial memory was assessed in trials performed on 5 consecutive days, and the time spent in the target quadrant after removing the platform was measured on the last day. DigBehav System was used to record and analyse the data.

### Tissue preparation

The animals were sacrificed by decapitation, and brain tissue was isolated. The hippocampus was harvested from the isolated brains and fixed in 4% paraformaldehyde. The remaining tissue was homogenized in phosphate buffer solution to assess biochemical parameters.

### Measurement of neuronal apoptosis

The TUNEL assay was performed to assess neuronal apoptosis (Dölle et al. 2018). The hippocampus was sectioned (5 µm thickness), and the DeadEnd™ fluorometric TUNEL System kit was used to estimate neuronal apoptosis. The nuclei were stained with Hoechst stain, and NIS-Elements Basic Research imaging software was used to determine the number of TUNEL-positive cells.

### Measurement of cytokine levels

The levels of the inflammatory mediators NF-kB, IL-1β, IL-6, and TNF-α were measured in brain tissue homogenates using ELISA per the kit protocols.

### Measurement of oxidative stress

A riboflavin-sensitized method was used to assess the activity of superoxide dismutase, and the absorbance was measured at 460 nm. The method reported by Ohkawa was used to determine the level of lipid peroxidation in brain tissues. The malondialdehyde level was determined in brain tissue at 523 nm wavelength.

### Western blotting

The expression of caspase-3, Bcl-2, Bax, TLR-4, cyclin B1, and Sirt-1 was assessed in isolated brain tissues using Western blotting. The protein content in tissue homogenates was determined using the BCA assay kit, and proteins were separated by 10% sodium dodecyl sulphate-polyacrylamide gel electrophoresis. The separated proteins were transferred to a membrane, which was then blocked in 5% blocking reagent. The membrane was incubated with primary antibodies targeting caspase-3 (9H19L2, 1:1000), Bcl-2 (MA5-11757; 1:500), Bax (MA5-14003; 1:500), TLR-4 (48-2300; 1:100), cyclin B1 (MA1-155; 1:100), and Sirt-1 (MA5-15677; 1:100) overnight at 4 °C. After washing with PBS, the membrane was incubated with goat secondary antibodies conjugated to horseradish peroxidase, and protein expression was detected using a chemiluminescence kit.

### qRT-PCR

SYBR green-based qRT-PCR was used to determine the mRNA expression of Sirt-1, TLR-4, NF-kB, and cyclin B1. Total RNA was extracted using TRIzol reagent and then subjected to TaqMan MicroRNA assays. Moloney murine leukaemia virus reverse transcriptase was used to synthesize cDNA from 2 µg total RNA (20 µL). The primers listed below were mixed with RT 2 SYBR Green Master Mix and added to the cDNA to determine gene expression using the Quantitative SYBR Green PCR assay. The relative target-gene expression levels were determined using the 2^−ΔΔCq^ method.

**Table:** 

Primers	Forward	Reverse
TLR-4	5′CCCTCATGACATCCCTT′ATTCA3′	5′CTGTCAGTACCAAGGTTGAGAGC3′
NF-κB	5′GAGCAAATGGTGAAGGAG3′	5′TCTGGAAGTTGAGGAAGG3′
Cyclin B1	5′GCAGCACCTGGCTAAGAATGT3′	5′GCCTTGGCTAAATCTTGAACT3′
SIRT-1	5′CAAGGAAATCTACCCCGGACAGT3′	5′CAGTGTGTCGATATTCTGCGTGT3′
β-actin	5′AGTGTGACGTTGACATCCGTAA3′	5′GGACAGTGAGGCCAGGATAGA3′

### Immunohistochemical analysis

The 3,3′-diaminobenzidine method was used to stain brain tissues, as described previously (Sun 2010). Isolated brain tissue was sectioned and incubated with Sirt-1 protein antibodies at 4 °C overnight. A BX51 light microscope was used to visualize the immunoreactive proteins, and the protein densities were estimated using Image-Pro Plus.

### Statistical analysis

All data are expressed as the means ± standard error of the mean (SEM; n = 15), and the data were compared using one-way analysis of variance. Post hoc comparisons were made using Dunnett’s test in GraphPad Prism (ver. 6.1; GraphPad Software, San Diego, CA, USA). In all analyses, *p* < 0.05 was taken to indicate statistical significance.

## Results

### DOP alleviates ISF-induced cognitive dysfunction

**Fig. 1:**
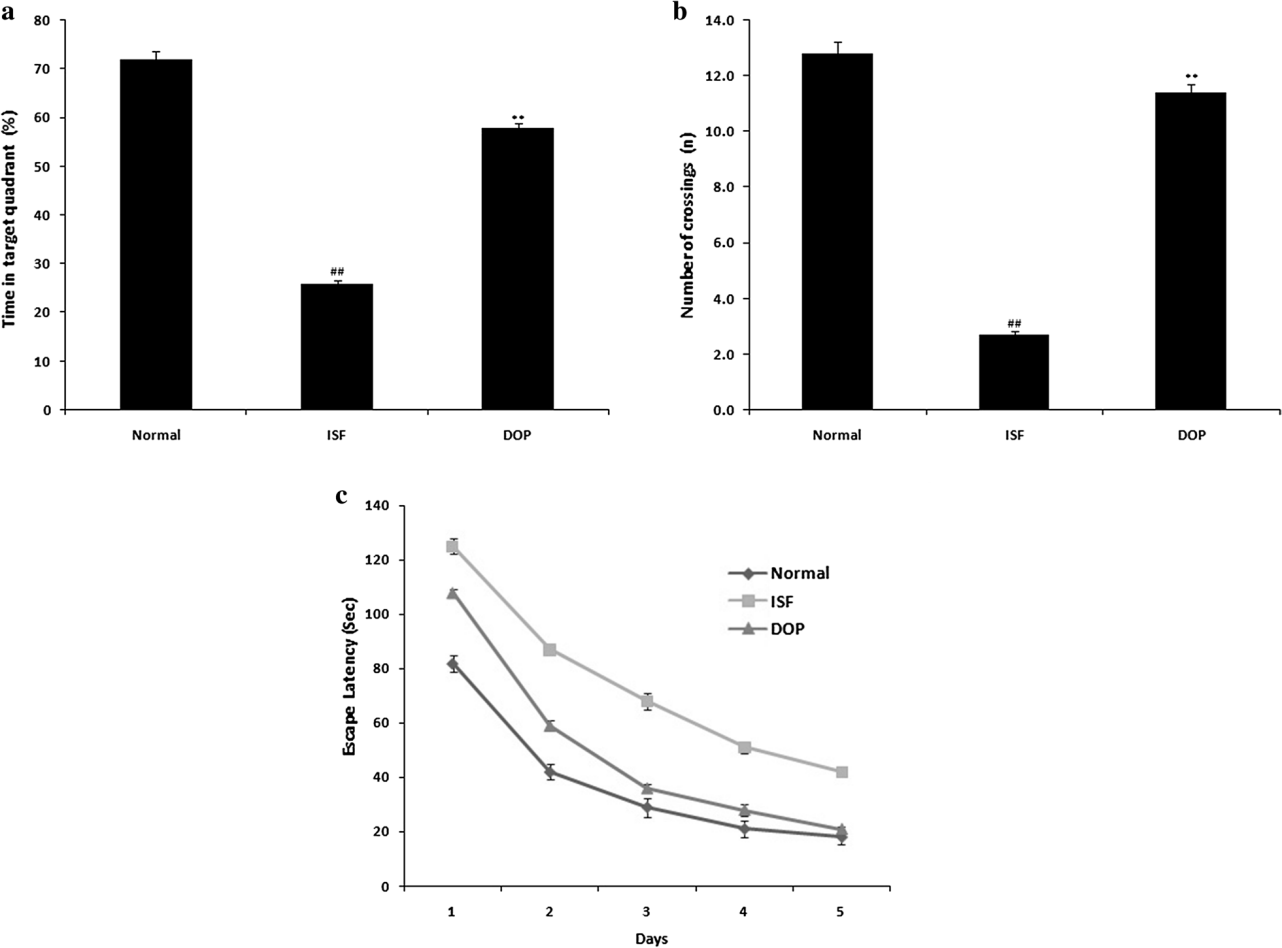
Desoxyrhapontigenin ameliorates the cognitive function of rats with anaesthesia-induced neuronal injury. **a** Time in target quadrant (%). **b** Number of crossings (n). **c** Escape latency(s). Mean ± SEM (n = 15); ^##^*p* < 0.01 vs. controls; **p < 0.01 vs. ISF group

Figure 1 shows the cognitive function of DOP- and ISF-treated rats based on the MWM. The number of crossings and time spent in the target quadrant were lower, and the escape latency higher, in the ISF group than control group. DOP treatment improved the spatial memory in rats with ISF-induced neuronal injury.

### DOP alleviates the ISF-induced increase in the neurological score

**Fig. 2:**
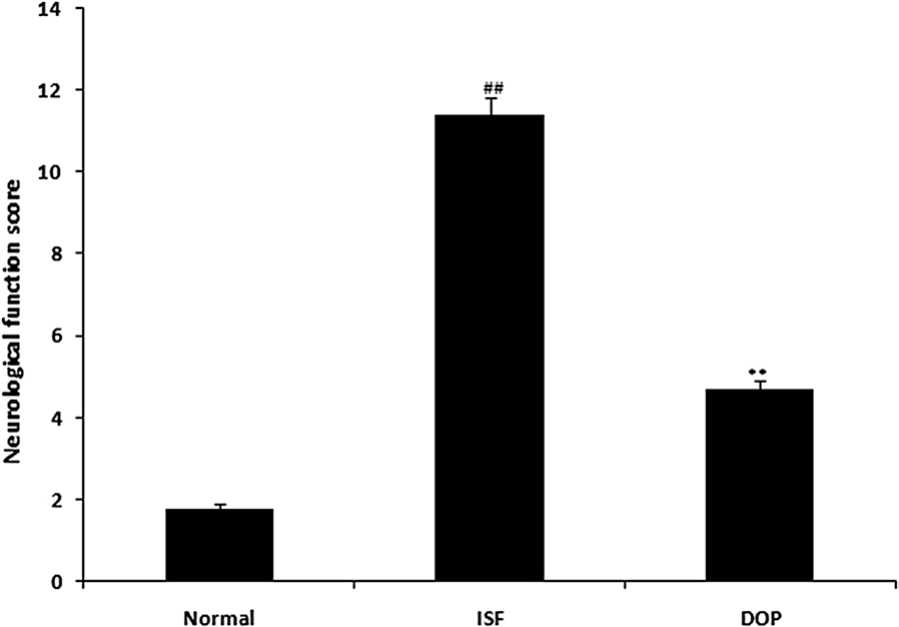
Desoxyrhapontigenin ameliorates the neurological score in rats with anaesthesia-induced neuronal injury. Mean ± SEM (n = 15); ^##^*p* < 0.01 vs. controls; **p < 0.01 vs. ISF group

The neurological function score was 11.4 in the ISF group compared with 1.8 in the control group (Fig. 2). The score was 4.7 in the DOP group.

### DOP alleviates ISF-induced neuronal apoptosis

**Fig. 3:**
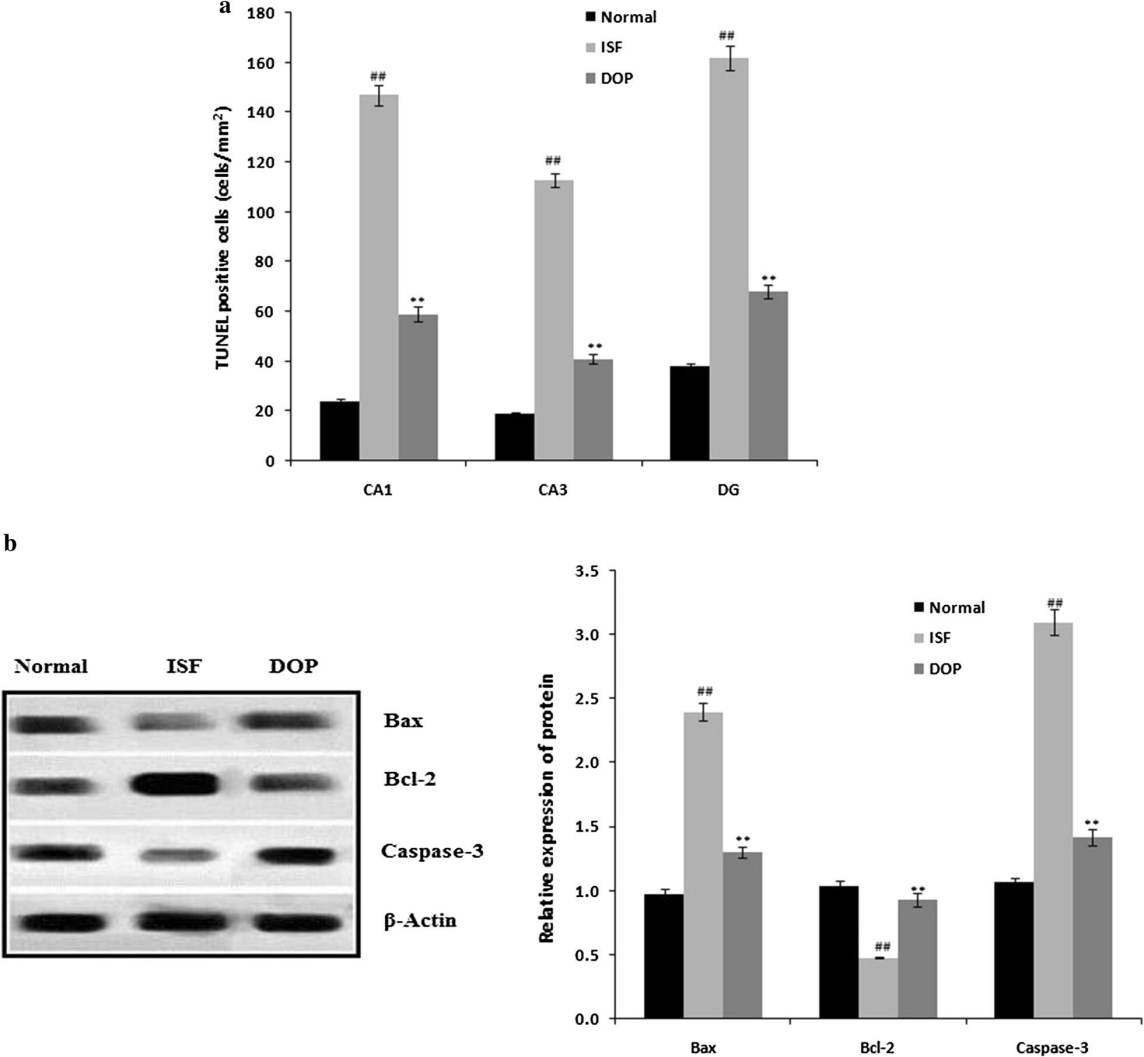
Desoxyrhapontigenin ameliorates neuronal apoptosis in the brain tissues of rats with anaesthesia-induced neuronal injury. **a** TUNEL-positive cells; **b** Protein expression of caspase-2, Bcl-2, and Bax by Western blotting. Mean ± SEM (n = 15); ^##^*p* < 0.01 vs. controls; **p < 0.01 vs. ISF group

Figure 3 shows the effect of DOP on neuronal apoptosis, based on the number of TUNEL-positive cells and protein expression of caspase-2, Bcl-2, and Bax in brain tissues with neuronal injury. The numbers of TUNEL-positive cells were higher in brain areas CA1, CA3, and DG in the ISF group compared with the control group. Treatment with DOP reduced the number of TUNEL-positive cells compared with the ISF group (Fig. 3a). In addition, the Bax and caspase-3 expression was enhanced, and the Bcl-2 expression reduced, in the brain tissue of the ISF group compared with the normal group. In the DOP group, the Bax and caspase-3 expression was reduced, and the Bcl-2 expression increased, compared with the ISF group (Fig. 3b).

### DOP reduces ISF-enhanced cytokine levels

**Fig. 4:**
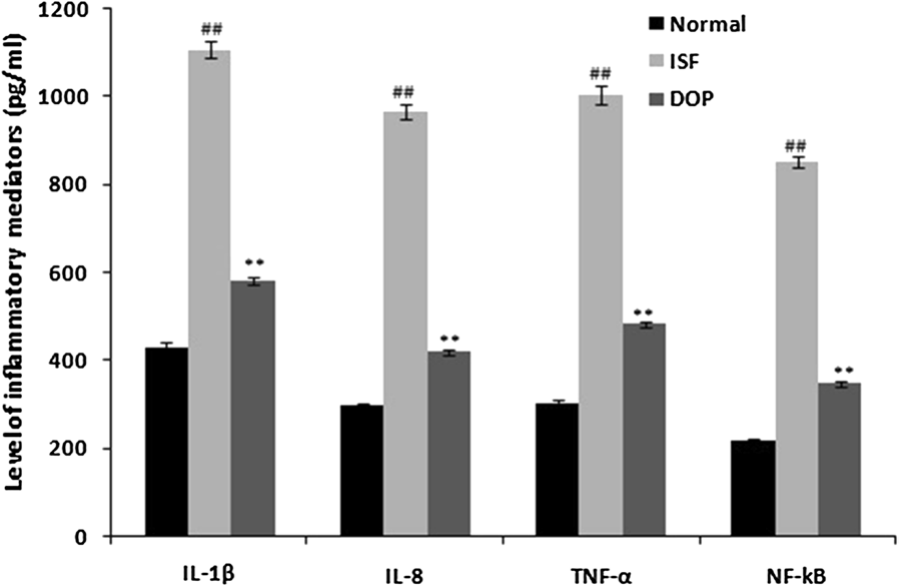
Desoxyrhapontigenin ameliorates the levels of cytokines in the brain tissues of rats with anaesthesia-induced neuronal injury. Mean ± SEM (n = 15); ^##^*p* < 0.01 vs. controls; **p < 0.01 vs. ISF group

The levels of inflammatory mediators were enhanced in the brains of the ISF group compared with the normal group and were reduced significantly in the DOP group compared with the ISF group (Fig. 4).

### DOP alleviates oxidative stress

**Fig. 5:**
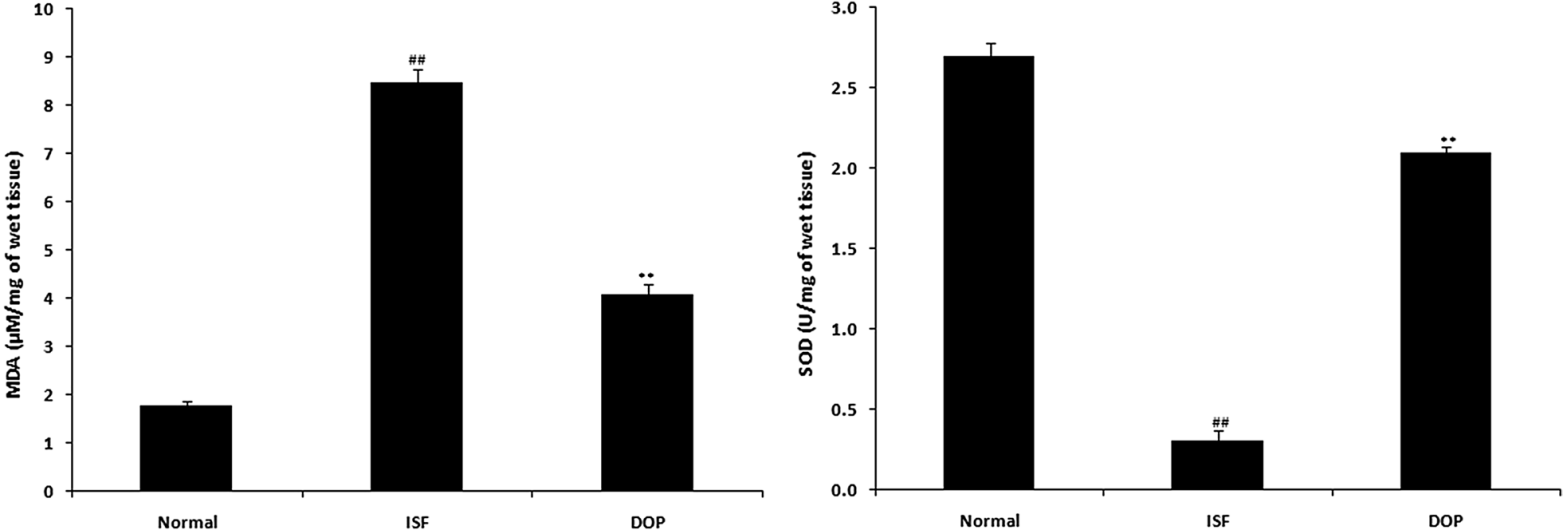
Desoxyrhapontigenin ameliorates the level of oxidative stress in the brain tissues of rats with anaesthesia-induced neuronal injury. Mean ± SEM (n = 15); ^##^*p* < 0.01 vs. controls; **p < 0.01 vs. ISF group

The malondialdehyde level was enhanced and superoxide dismutase activity reduced in the brain homogenates of the ISF group compared with the control group; however, DOP treatment attenuated the level of oxidative stress (Fig. 5).

### DOP alleviates the ISF-induced alterations in TLR-4, cyclin B1, and Sirt-1 protein expression

**Fig. 6:**
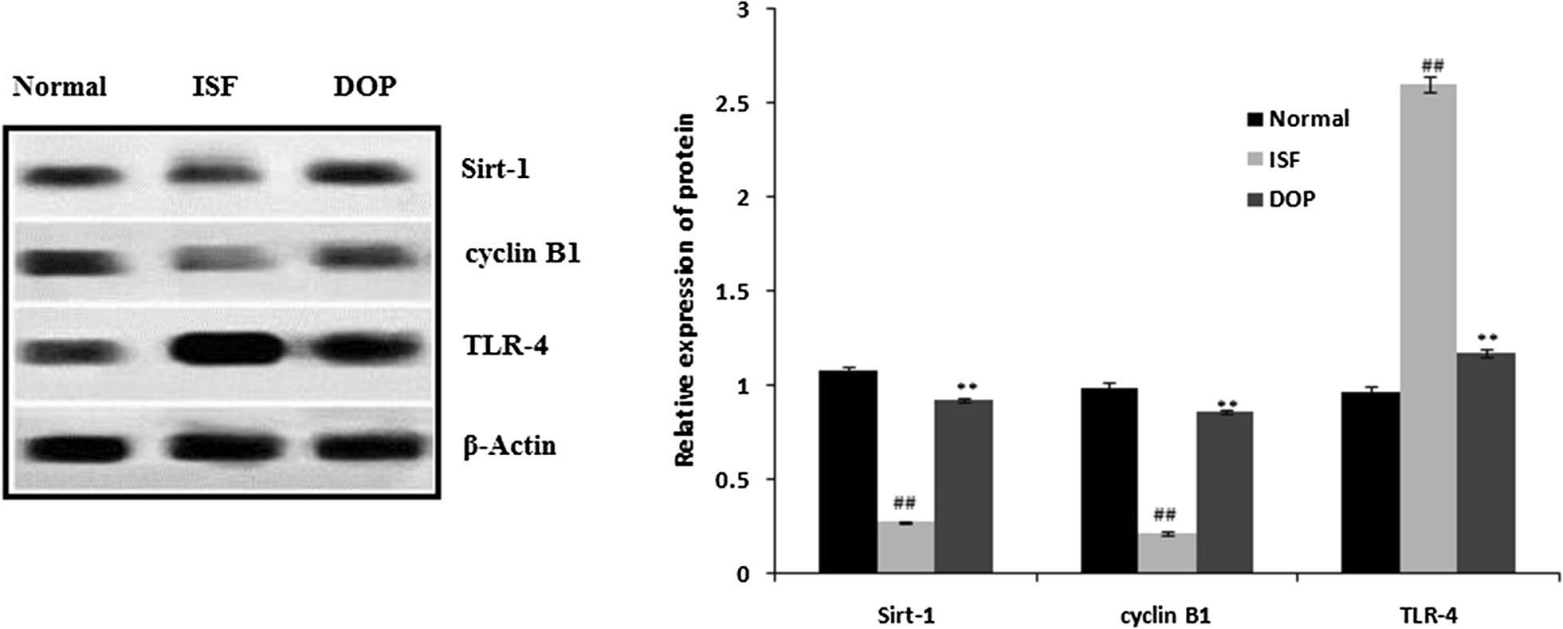
Desoxyrhapontigenin ameliorates the protein expression of TLR-4, cyclin B1, and Sirt-1 in the brain tissues of rats with anaesthesia-induced neuronal injury. Mean ± SEM (n = 15); ^##^*p* < 0.01 vs. controls; **p < 0.01 vs. ISF group

According to Western blotting, the protein expression of cyclin B1 and Sirt-1 was reduced and TLR-4 increased in the tissue homogenates of the ISF group compared with the controls (Fig. 6). DOP treatment attenuated these changes.

### DOP alleviates the ISF-induced alterations in TLR-4, NF-kB, cyclin B1, and Sirt-1 mRNA expression

**Fig. 7:**
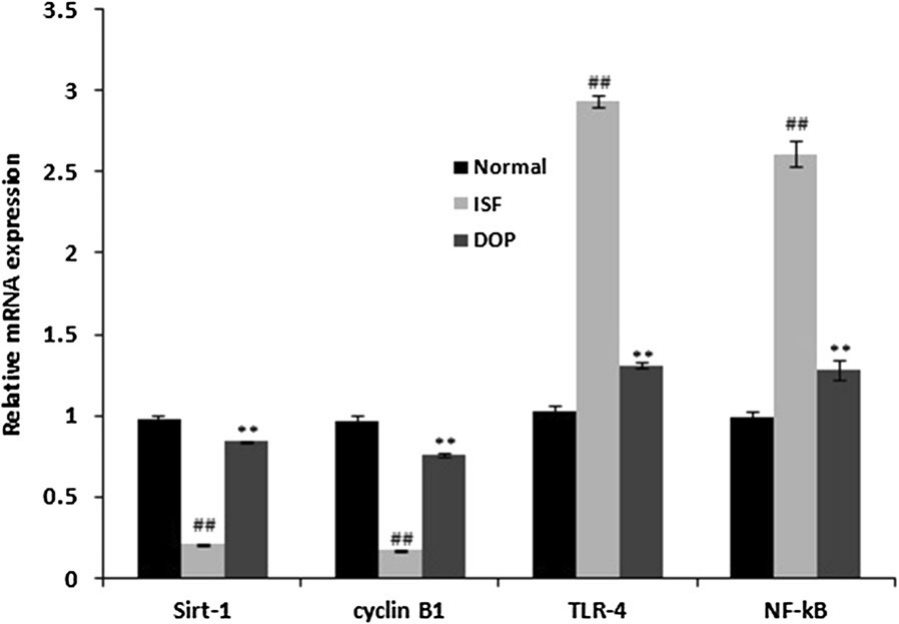
Desoxyrhapontigenin ameliorates the mRNA expression of TLR-4, NF-kB, cyclin B1, and Sirt-1 in the brain tissues of rats with anaesthesia-induced neuronal injury. Mean ± SEM (n = 15); ^##^*p* < 0.01 vs. controls; **p < 0.01 vs. ISF group

According to qRT-PCR, ISF reduced cyclin B1 and Sirt-1 mRNA expression and increased TLR-4 and NF-kB mRNA expression in the brain homogenates of rats with ISF-induced neuronal injury compared with the controls (Fig. 7). DOP ameliorated these changes.

### DOP alleviates the ISF-induced reduction in Sirt-1 protein expression

**Fig. 8:**
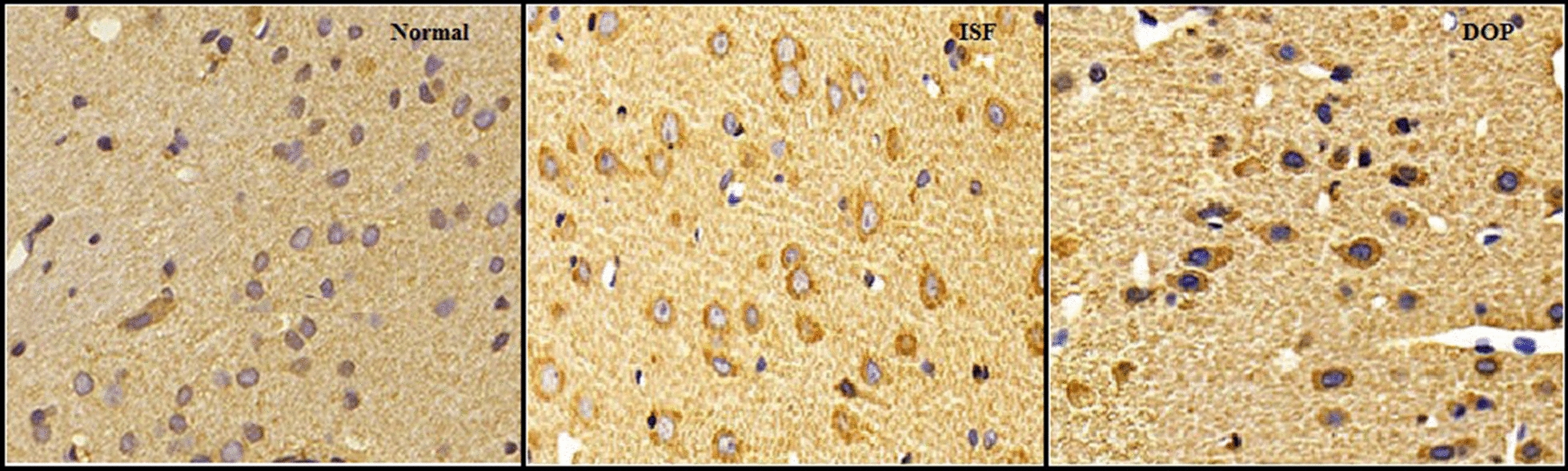
Desoxyrhapontigenin ameliorates the protein expression of Sirt-1, according to immunohistochemical analysis, in the brain tissues of rats with anaesthesia-induced neuronal injury

Sirt-1 immunohistochemical protein expression was reduced in the brain homogenates of the ISF group compared with the control group (Fig. 8). DOP treatment attenuated this change.

## Discussion

Anaesthetics like isoflurane are commonly used for the image studies and surgical procedure including in children (Bodolea 2016). Prolonged exposure of isoflurane in the neonatal rats causes defects in cognitive function due to induction of neurodegenration (Xu et al. 2015). Neuronal function ability such as writing, reading and learning reported to be reduced in the children with less than 4 years of age exposed to anaesthetics (Isoflurane) (Seubert et al. 2013). However reported studies suggested that isoflurane has shown higher incidences of neuronal apoptosis than sevoflurane in neonatal rats (Lei et al. 2012). Literature reveals that isoflurane causes damages several regions of brain including hippocampus, prefrontal cortex and thalamus by inducing neuronal apoptosis (Peng et al. 2014). In animals exposed to ISF, neurons are damaged by activation of apoptosis (Burchell et al. 2013), as supported by our findings. However, DOP treatment reversed the altered cognitive function and reduced neuronal apoptosis induced by exposure to the anaesthetic ISF in rats. DOP treatment attenuated the expression of caspase-3, Bax, and Bcl-2 in the brain homogenates of rats with neuronal injury.

ISF exposure in the developing brain increases oxidative stress, which leads to further neuronal injury as it enhances inflammation (Safavynia and Goldstein 2019). In our study, the level of oxidative stress was reduced in the DOP group compared with the ISF group. Inflammatory mediators are also involved in the cognitive defects and neuronal injury induced by ISF in developing brains (Kawasaki and Kawai 2014). These mediators enhance the inflammatory process by activating NF-κB signalling. Inflammatory cytokine levels were significantly reduced (*p* < 0.01) in the brain tissue of the DOP group compared with the ISF group. The transmembrane protein TLR4 regulates innate immunity by activating inflammatory cytokines and NF-kB signalling (Aliprantis et al. 2000). TLR-4 activation contributes to neuronal injury by regulating pro- and anti-apoptotic proteins (Fujita and Yamashita 2018). Controlling TLR-4 signalling protects against neuronal apoptosis, and our results suggest that DOP treatment ameliorates the altered TLR-4 signalling in neuronally injured rats.

In the developing brain, the expression of Sirt-1 is enhanced, and its activation prevents neuronal injury (Xu et al. 2018). Sirt-1 regulates the mitochondrial apoptosis pathway. Inflammatory mediators reduce the protein expression of Sirt-1 in the brain, and our data showed that DOP treatment reversed the expression of Sirt-1 protein in injured neuronal tissues.

In conclusion, DOP ameliorates the alterations in neuronal apoptosis and cognitive functions in rats with ISF-induced neuronal injury. Moreover, DOP treatment prevents neuronal injury by regulating the TLR-4/cyclin B1/Sirt-1 pathway.

## Data Availability

The supporting data for present findings is under ethics restrictions and is hence not presented here.
